# Complete Genome Sequence of a Genotype 2B Rubella Virus Isolated in South Korea in 2015

**DOI:** 10.1128/genomeA.00940-17

**Published:** 2017-09-21

**Authors:** Hae Ji Kang, You-Jin Kim, Hye Min Lee, Jeong-Gu Nam, Sung Soon Kim

**Affiliations:** Division of Respiratory Viruses, Center for Infectious Diseases, National Institute of Health, Korea Centers for Disease Control and Prevention, Cheongju-si, Chungbuk, South Korea

## Abstract

The complete genome sequence of the wild-type genotype 2B rubella virus RVi/Busan.KOR/10.15[2B], isolated from a patient in South Korea, was determined. The availability of this sequence will help in understanding the circulation of endemic rubella viruses, as well as their genetic diversity.

## GENOME ANNOUNCEMENT

Rubella is a highly contagious disease that is commonly considered to be a mild rash illness, but it remains an important global public health problem because it can lead to miscarriage or congenital rubella syndrome in the early stages of pregnancy (1, 2).

Effective vaccines and routine immunization programs have reduced the number of rubella cases in many countries (3). In South Korea, the monovalent rubella vaccine was introduced in 1978, and the measles, mumps, and rubella (MMR) vaccine was added to the national immunization program in 1983. A two-dose MMR vaccination schedule was implemented in 1997 (4). Because of such national efforts, the average annual number of rubella cases has been maintained at 30 since 2001, based on data from the national infectious diseases surveillance system operated by the Korea Centers for Disease Control and Prevention (KCDC).

Rubella is caused by the rubella virus, which is the only member of the *Rubivirus* genus in the *Togaviridae* family. Rubella virus has a single-stranded positive-sense RNA genome with 9,762 nucleotides encoding two nonstructural (P90 and P150) and three structural (one nucleocapsid and two envelope [E1 and E2]) proteins. Phylogenetic analysis based on nucleic acid sequences in the E1 coding region of the virus revealed two distinct groups, clades I and II. Ten genotypes (1a, 1B, 1C, 1D, 1E, 1F, 1G, 1H, 1I, and 1J) have been recognized as belonging to clade I, and three genotypes (2A, 2B, and 2C) have been recognized as belonging to clade II. Genotypes 1E and 2B are frequently detected and show global distribution (5, 6). Despite recent developments in whole-genome sequencing technologies, the number of available complete genome sequences of rubella virus is still less than 50 in GenBank. Among these sequences are only 13 complete genome sequences of genotype 2B isolates (6).

Here, we report the full-length genome sequence of a genotype 2B rubella virus isolated from a throat swab specimen of a 36-year-old male in Busan, South Korea, in March 2015 using Vero-hSLAM cells. The cultivated virus was deposited into the National Culture Collection for Pathogens (no. R2017F014), operated by the Korea National Research Institute of Health in South Korea. Total RNA was extracted from the infected Vero-hSLAM cells using the Qiagen RNeasy minikit (Qiagen) and reverse transcribed using SuperScript III first-strand synthesis systems (Invitrogen) with random hexamers. The viral genome was amplified by PCR using gene-specific primers that were designed based on previously published rubella virus sequences. Nucleotide sequences were obtained by direct sequencing using an ABI3730XL DNA analyzer (GnC Bio Co., South Korea). The complete genome of rubella virus strain RVi/Busan.KOR/10.15[2B] contained 9,761 nucleotides, and the virus was phylogenetically classified as genotype 2B. The genome showed 99.2% nucleotide sequence similarity with the complete genome of RVi/HochiMinh.VNM/20.12/Rvv129[2B] (GenBank accession no. AB928203) and 96.3%, 94%, and 97.1% identities with the structural protein gene sequences of the WHO reference strains RVi/Anhui.CHN/00/2[2B] (AY968218), RVi/TelAviv.ISR/68[2B] (AY968219), and RVi/Wash.USA/16.00[2B] (AY968220), respectively.

### Accession number(s).

This whole-genome shotgun project for the RVi/Busan.KOR/10.15[2B] isolate has been deposited in GenBank under the accession no. MF496142.
